# Supplementary material for: Deformation constraints of graphene oxide nanochannels under reverse osmosis
Source: Nat Commun. 2023 Feb 23;14:1016. doi: 10.1038/s41467-023-36716-5 (PMC9950365; doi:10.1038/s41467-023-36716-5)
Supplement: Supplementary file 1 — Supplementary Information [file 41467_2023_36716_MOESM1_ESM.pdf]

SUPPLEMENTARY INFORMATION

Deformation constraints of graphene oxide nanochannels under reverse osmosis

Kecheng Guan[1], Yanan Guo[2], Zhan Li[1], Yuandong Jia[1,3], Qin Shen[1,3], Keizo Nakagawa[1,4], Tomohisa Yoshioka[1,4], Gongping Liu[2], Wanqin Jin[2,*], Hideto Matsuyama[1,3,*]

1. Research Center for Membrane and Film Technology, Kobe University, 1-1 Rokkodai, Nada, Kobe 657-8501, Japan
2. State Key Laboratory of Materials-Oriented Chemical Engineering, College of Chemical Engineering, Nanjing Tech University, 30 Puzhu Road (S), Nanjing 211816, China
3. Department of Chemical Science and Engineering, Kobe University, 1-1 Rokkodai, Nada, Kobe 657-8501, Japan
4. Graduate School of Science, Technology and Innovation, Kobe University, 1-1 Rokkodai, Nada, Kobe 657-8501, Japan

*Corresponding authors: wqjin@njtech.edu.cn (W. Jin); matuyama@kobe-u.ac.jp (H. Matsuyama)

This PDF file includes:

Supplementary Notes
Supplementary Figures 1 to 23
Supplementary Tables 1 to 3
Supplementary References
Legends for Supplementary Movies 1 to 6

Other supporting materials for this manuscript include the following:

Supplementary Movies 1 to 6

Supplementary Notes

Materials

A commercial aqueous suspension of GO (2 mg mL^{-1}) was purchased from Sigma-Aldrich Co., LLC (St. Louis, MO, USA). D(+)-Glucose (Glc) and 25% aqueous ammonia solution were purchased from FUJIFILM Wako Pure Chemical Industries Co., Ltd. (Osaka, Japan), and employed to reduce GO. Commercial nylon (pore size = 0.2 μm; hydrophilic; diameter = 47 mm) and polycarbonate (pore size = 0.2 μm; hydrophilic; diameter = 47 mm) membranes were purchased from Merck Millipore (Burlington, VT, USA) and were used as substrates to support the GO or Glc-rGO laminates. Additionally, salts (Na_2SO_4, NaCl, $MgSO_4$, and $MgCl_2$) and carbohydrates (glycerol, D(+)-Glc, sucrose, and D(+)-raffinose pentahydrate) were purchased from FUJIFILM Wako Pure Chemical Industries Co., Ltd. (Osaka, Japan), for the preparation of feed solutions for rejection tests and MWCO analyses, respectively. Ultrapure water (Milli-Q Integral 3, Millipore SAS, Molsheim, France) was used for all experiments. All the chemicals were used without further purification.

Supplementary figures

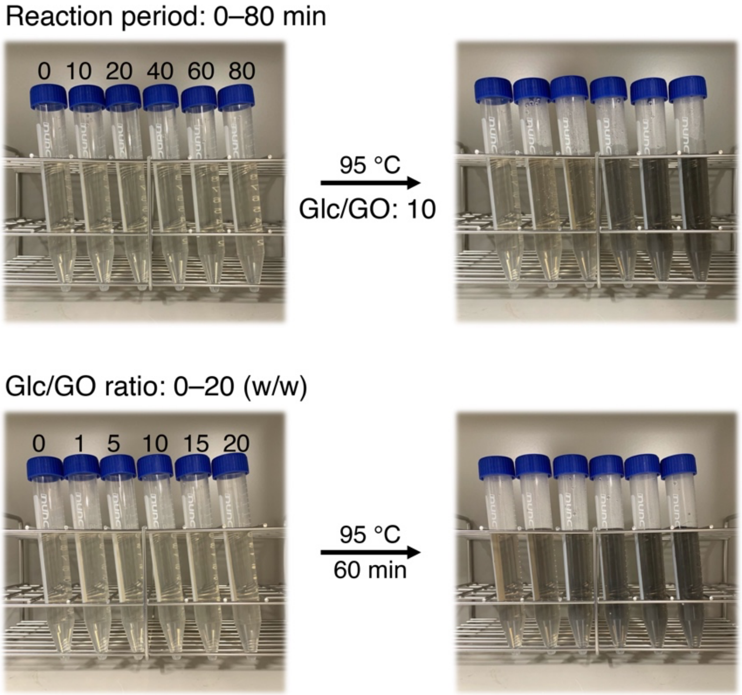

Supplementary Fig. 1. Digital images of GO suspensions before and after chemical reactions with varying reaction periods and Glc/GO weight ratios. For the study on the effect of reaction period, the Glc/GO ratio was fixed at 10; For the study on the effect of Glc/GO ratio, the reaction period was fixed at 60 min.

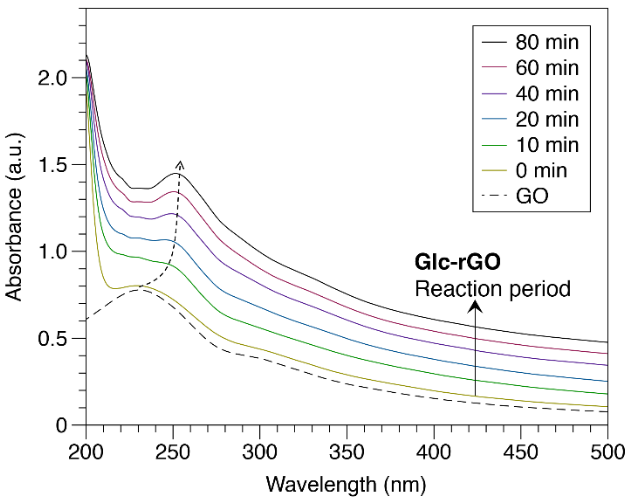

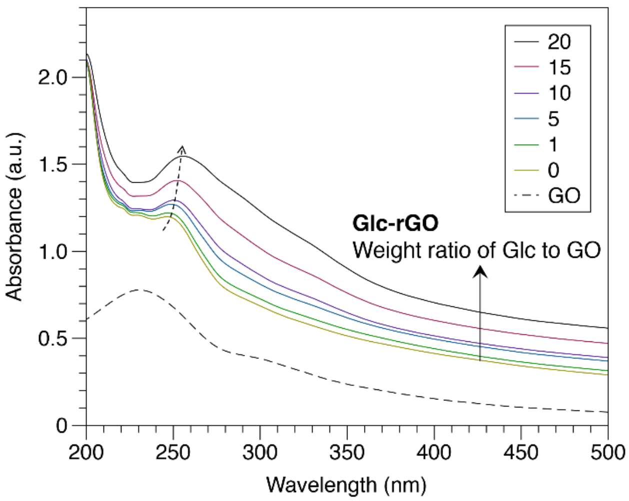

Supplementary Fig. 2. UV-vis spectra of the GO suspensions after chemical reactions. A red shift of the GO characteristic peak was observed when the reaction period and Glc ratio were increased, indicating a higher degree of reduction of GO.

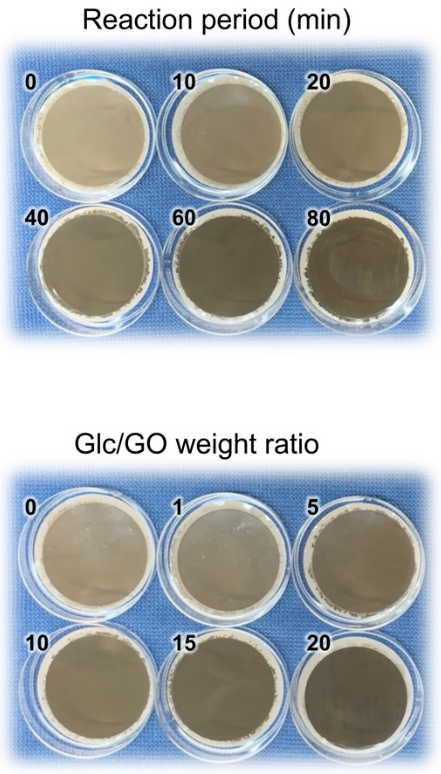

Supplementary Fig. 3. Digital images of deposited GO laminates on substrate membranes using the produced suspensions with varied reaction conditions of reaction period and Glc/GO ratio.

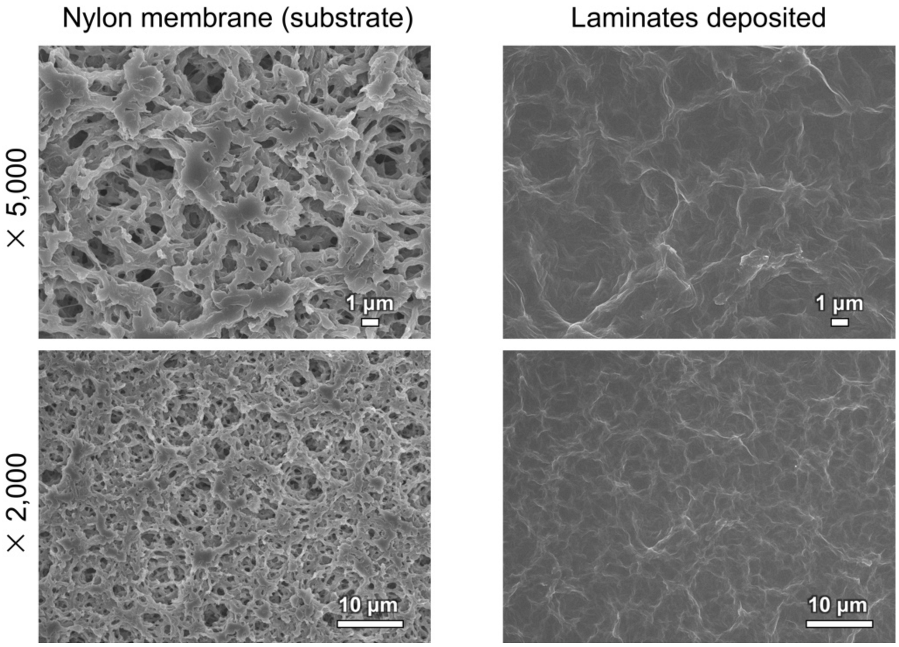

Supplementary Fig. 4. SEM images with different magnifications exhibiting the surface morphologies of porous nylon membranes before and after the deposition of a typical laminate membrane layer (Glc-rGO; reaction period, 60 min; and Glc/GO ratio, 10).

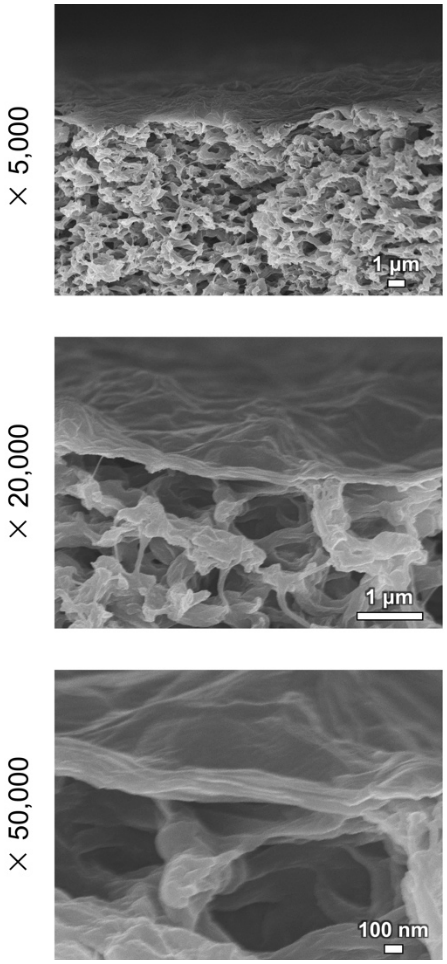

Supplementary Fig. 5. SEM images with different magnifications exhibiting the cross-section morphology of the laminates-deposited nylon membranes (Glc-rGO; reaction period, 60 min; Glc/GO ratio, 10).

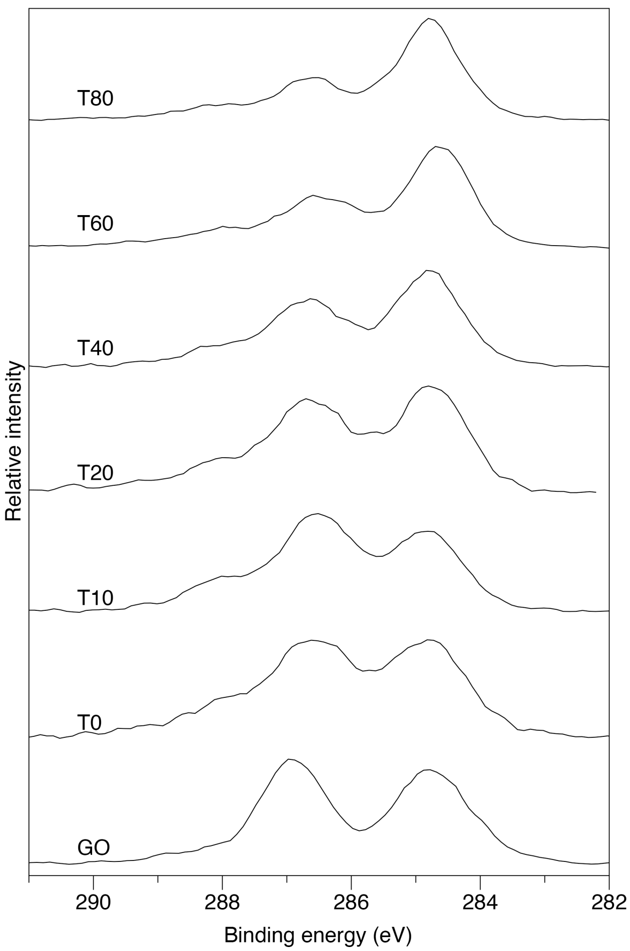

Supplementary Fig. 6. XPS C1*s* spectra of GO and Glc-rGO (varying reaction periods) laminates. The peak in the binding energy range of 286–289 eV exhibits a decreasing intensity with an increasing reaction period, indicating the loss of oxygenated functional groups.

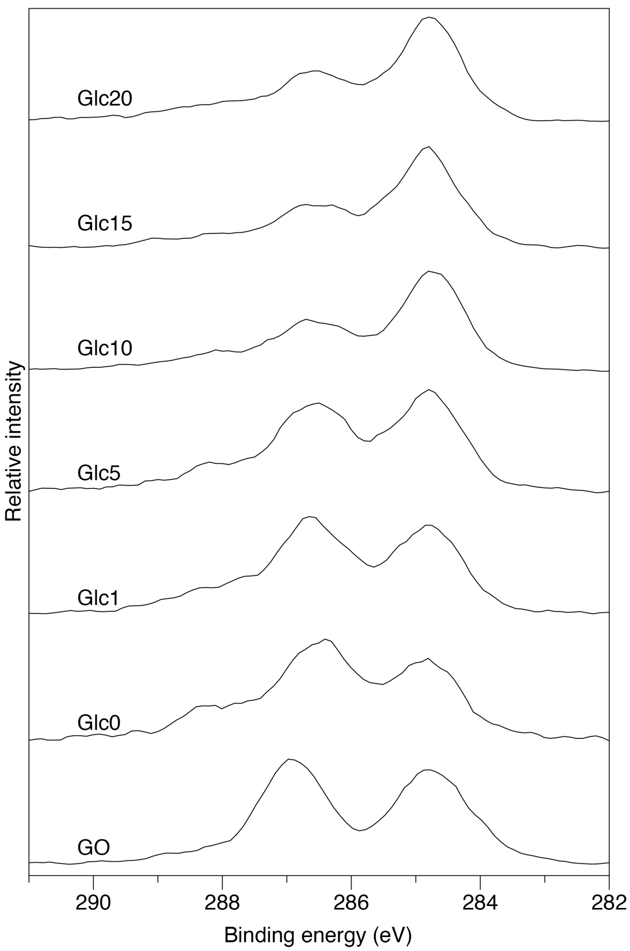

Supplementary Fig. 7. XPS C1*s* spectra of GO and Glc-rGO (varying Glc ratio) laminates. The peak in the binding energy range of 286–289 eV exhibits a decreasing intensity with an increasing Glc ratio, indicating the loss of oxygenated functional groups.

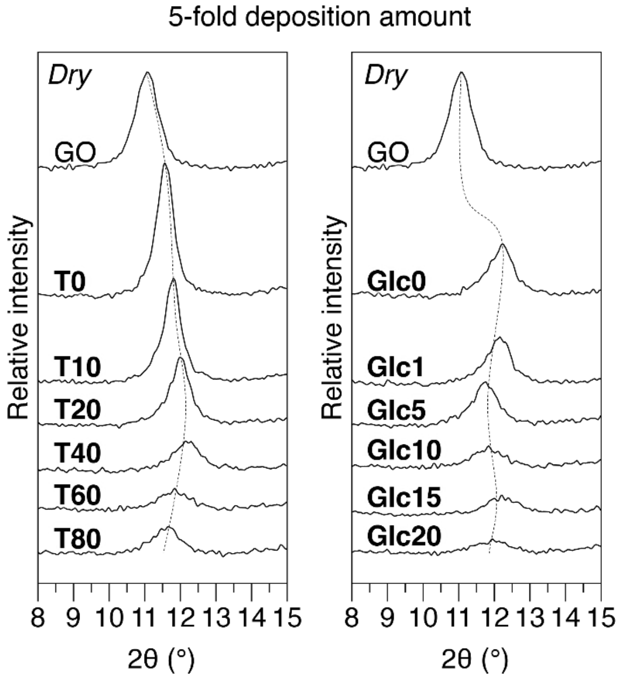

Supplementary Fig. 8. XRD spectra of thicker GO and Glc-rGO samples (dashed lines and arrows indicate peak shifts). These thicker samples were prepared by using 5-fold GO or Glc-rGO deposition amounts of normal samples.

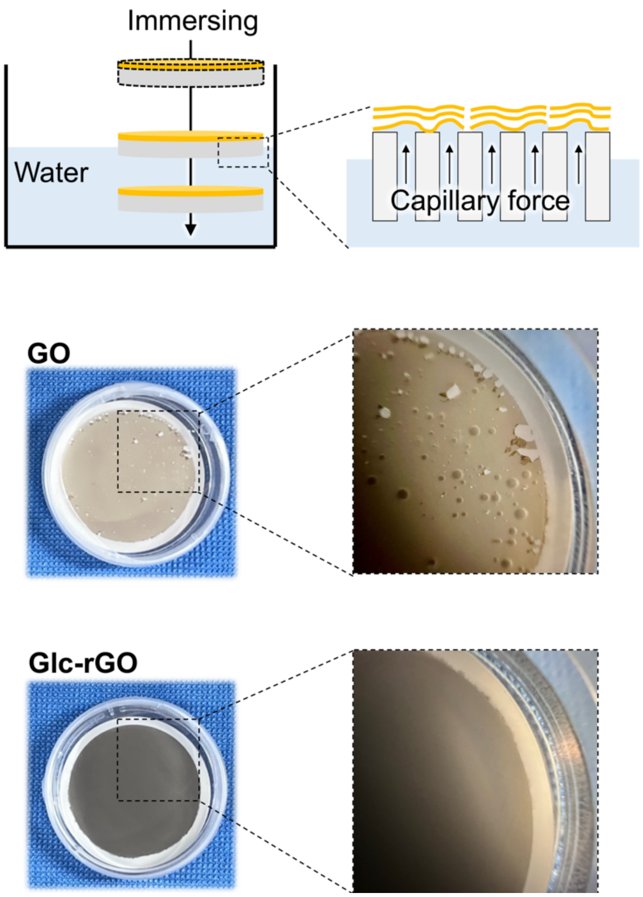

Supplementary Fig. 9. Macroscopic stability of laminate layer with water intrusion. Illustration of the water intrusion towards the laminates during immersion and surface morphology of GO and Glc-rGO (Glc10T60) after immersion.

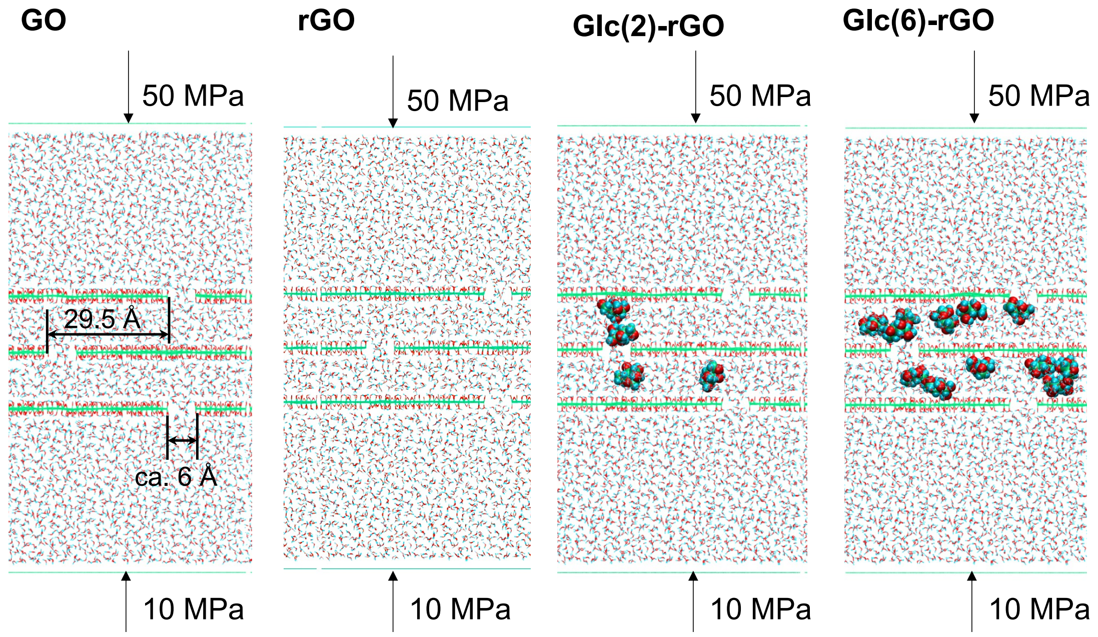

Supplementary Fig. 10. Constructed models to simulate laminate structure evolution in water with applied pressure applied. Slit offset, 29.5 Å; and slit size, ~6 Å for the model.

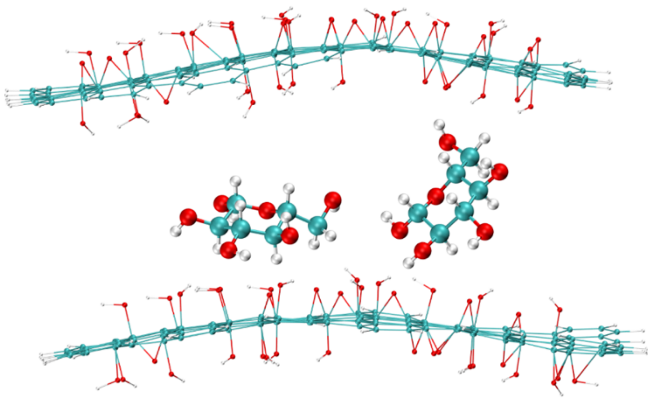

Supplementary Fig. 11. Glc-in-rGO channel model from the last frame of the MD simulation trajectory used for the IGM analysis.

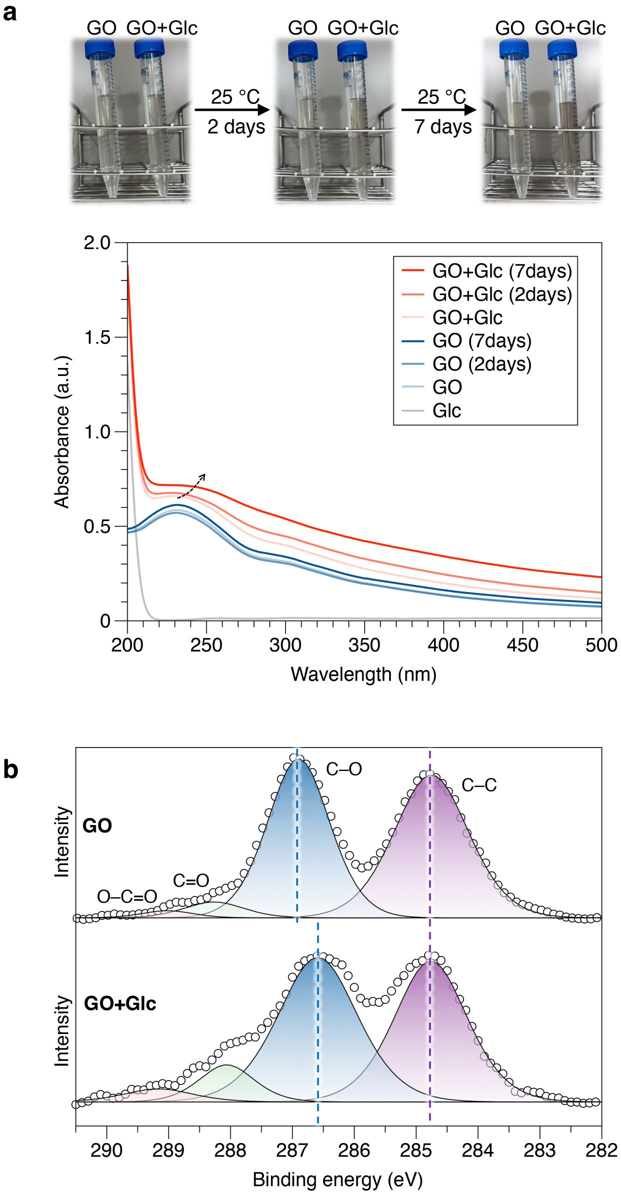

Supplementary Fig. 12. Characterizations of chemical affinity and interaction between GO and Glc. (a) Digital images showing suspensions and corresponding UV-vis spectra of GO (0.015 mg L^{-1}) and mixture of GO (0.015 mg L^{-1}) and Glc (0.15 mg L^{-1}); (b) XPS C1*s* spectra of GO and mixture of GO and Glc.

The suspension color of GO and Glc mixture (GO+Glc) became darker after a certain period even under room temperature (25°C), with observed higher overall absorbance and peak shift due to the chemical affinity of GO and Glc that leads to the chemical reduction of GO; whereas that of GO was not significantly changed. In addition, there was a binding energy shift for C–O bond of GO after the Glc mixing from XPS C1*s* spectra, indicating the interaction of hydrogen bonding between them with corresponding change of the chemical environment around the elements.

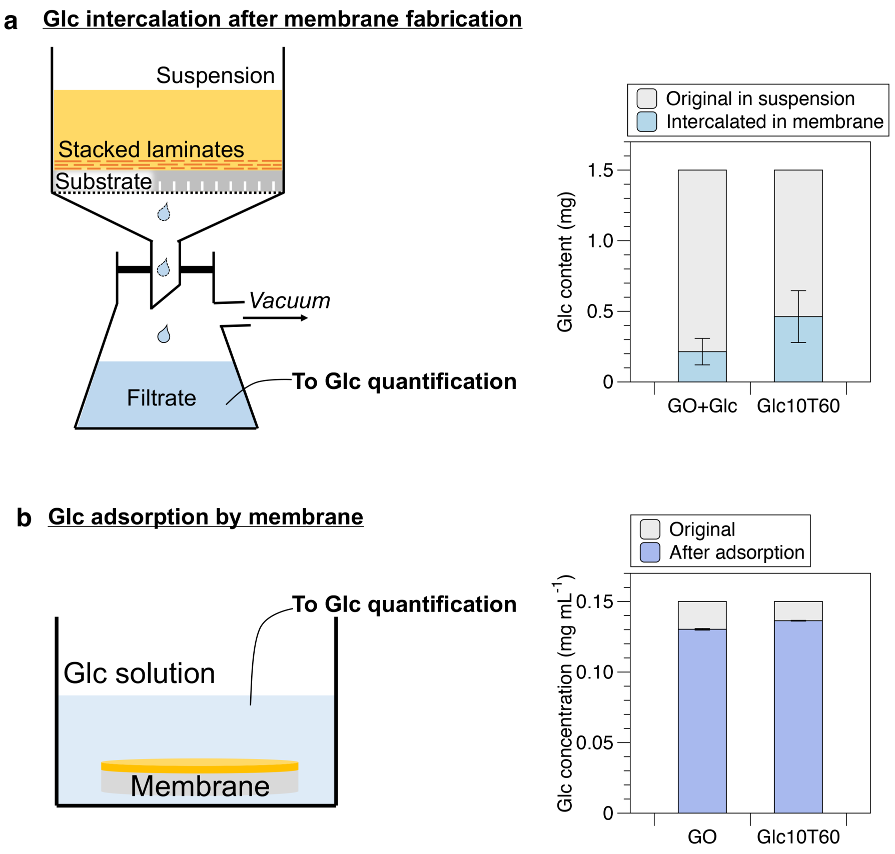

Supplementary Fig. 13. Glc intercalation in laminates and Glc adsorption by membranes. (a) Illustration of laminate fabrication with the filtration of suspensions of GO+Glc mixture and Glc10T60 and correspondingly quantified intercalation amount of Glc; (b) Illustration of Glc adsorption by GO and Glc10T60 membranes and correspondingly analyzed concentration change of Glc solution.

To measure the amount of Glc intercalated in the stacked laminates, the filtrate was collected after vacuum filtration of the suspensions (chemically converted Glc10T60 and simple mixture of GO and Glc) with a known amount of added Glc for the preparation of the laminates. The filtrate was analyzed using the total organic carbon (TOC) technique to quantify the Glc. Hence, the amount of Glc intercalated in the laminates was obtained. Control samples of the pristine GO suspension (without Glc addition) were also prepared to exclude the TOC from the filtered impurities of materials other than Glc.

To measure the adsorption of Glc by GO-based membranes, the membrane was immersed in a 10 mL Glc solution with an initial concentration of 0.15 mg mL^{-1}. After 2 days, the Glc solution concentration was measured by TOC. Control samples of bare substrate were also analyzed to exclude the adsorption by the substrate.

Glc could be intercalated in both GO laminates and chemically converted GO laminates. More Glc molecules could be retained in laminates possibly because of the chemical conversion process than that of simple mixture. GO membrane and Glc10T60 membrane could adsorb certain Glc from the solution. These results support the favorable interaction or affinity between GO and Glc.

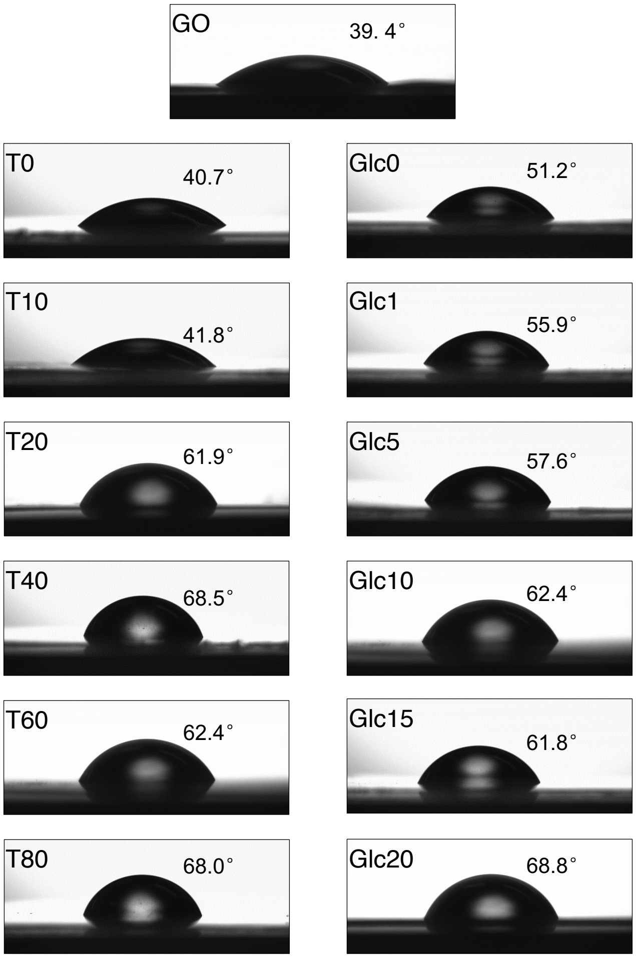

Supplementary Fig. 14. Water contact angle in air on the surfaces of GO and different Glc-rGO membranes.

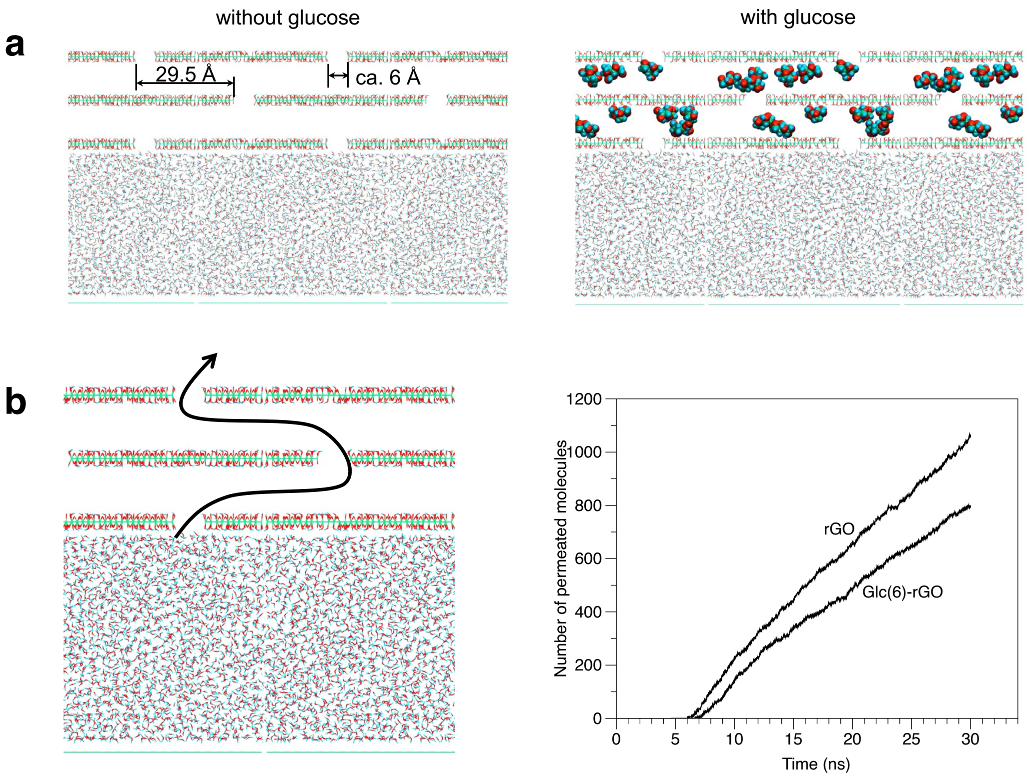

Supplementary Fig. 15. (a) Constructed models of GO and Glc(6)-rGO for simulation of water molecule permeation; (b) Number of water molecules permeated into the laminates. Slit offset, 29.5 Å; and slit size, ~6 Å for the model.

Pristine GO

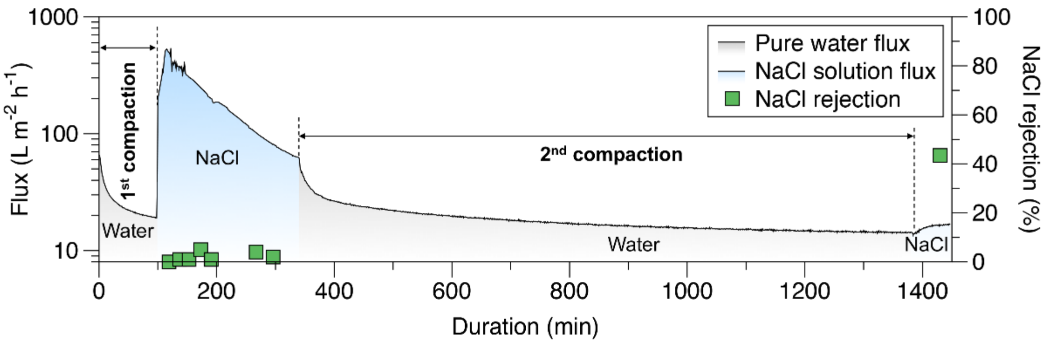

Supplementary Fig. 16. Filtration performance of the pristine GO membrane with different pre-compaction periods. Filtrations of pure water and NaCl solution (500 ppm) were conducted under 1 MPa. Membranes typically need to be compacted before use. Here, after compaction for approximately 100 min by pure water filtration (first compaction), the pristine GO membrane still exhibited significant instability towards the filtration of the NaCl solution, which was revealed by the gradual decline in permeation flux and the nearly 0% rejection. Therefore, we extended the compaction period to approximately16 h (second compaction), after which we observed that the NaCl rejection was increased to >40%. Thus, the pre-compaction period of 16 h was applied for all the membranes before any other following performance evaluations.

The duration of the first compaction process was too short to completely compact the GO membrane as the water flux of the GO membrane continued to decrease at a high rate. While the GO membrane was not completely compacted, changing the feed water to NaCl solution after the first compaction caused severe swelling of the GO membrane (a more than 20-fold flux increase), resulting a nearly 0% rejection. Therefore, in the second compaction process, the duration was prolonged until the flux decline of the GO membrane was minimized. In this case, the GO membrane was significantly more compacted than that after the first compaction process. It can be observed that changing the feed water to the NaCl solution after the second compaction process did not cause significant swelling of the GO membrane (a slight flux increase). Hence, the GO membrane after this sufficient compaction, the membrane exhibited moderate rejection of NaCl.

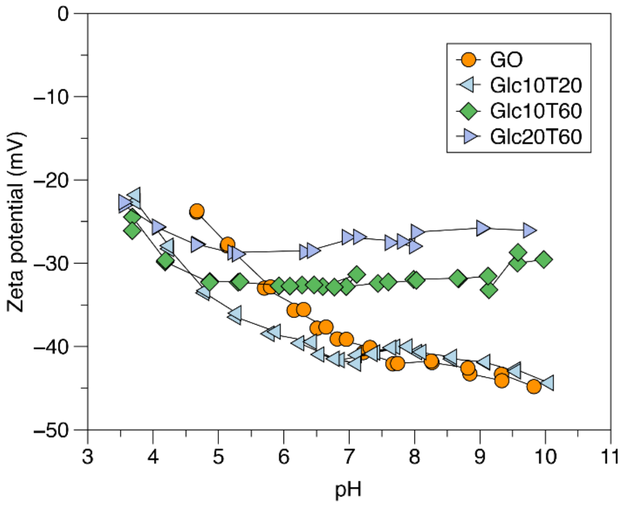

Supplementary Fig. 17. Surface zeta potential of GO, Glc10T20, Glc10T60, and Glc20T60 membranes in the pH range of ~3.5–10.

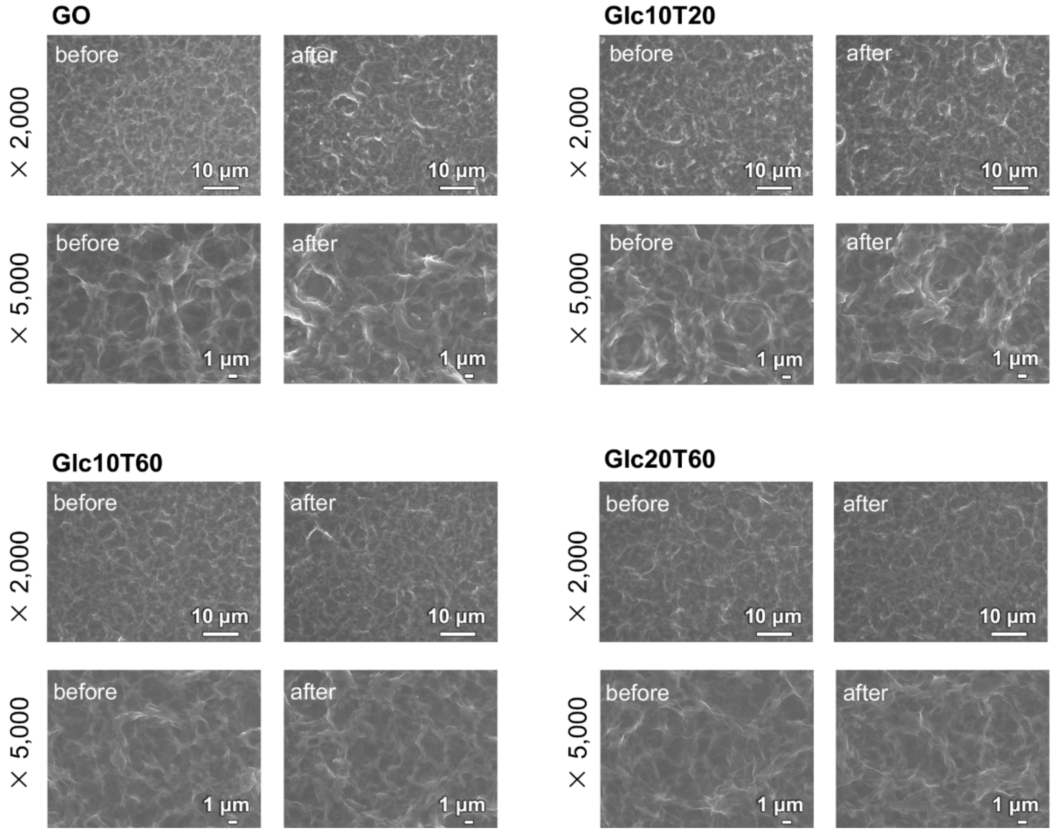

Supplementary Fig. 18. SEM images with different magnifications exhibiting the surface morphologies of the GO, Glc10T20, Glc10T60, and Glc20T60 membranes before and after pressurized filtrations.

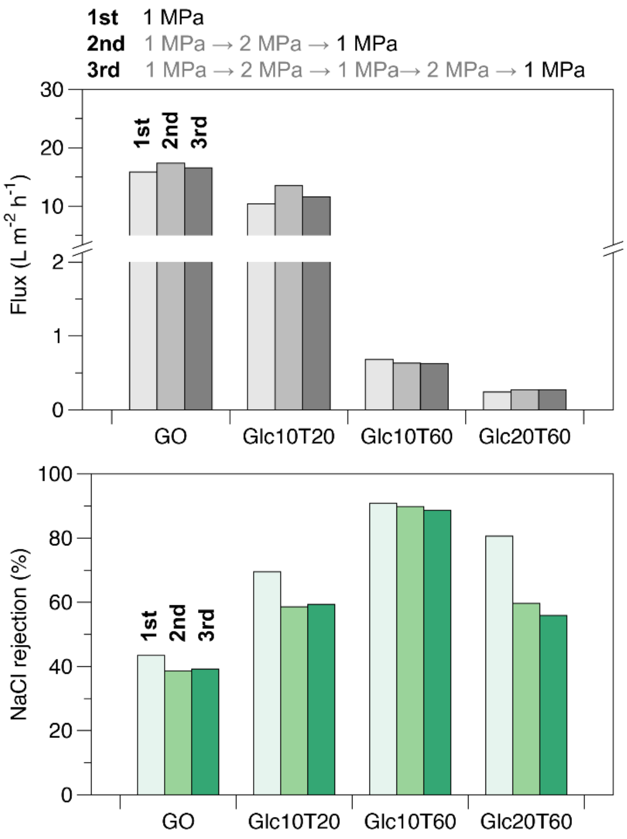

Supplementary Fig. 19. Average permeation flux and NaCl rejection of different membranes at three 1 MPa steps during pressure-changing reverse osmosis. The first filtration at 1 MPa was the initial test, whereas the second and third filtrations were tested after the filtration at 2 MPa.

The flux of these membranes at different steps of 1 MPa was similar, whereas the NaCl rejection was compromised after the first pressurization process for the GO, Glc10T20, and Glc20T60 membranes. The Glc10T60 membrane exhibited optimal stability and similar NaCl rejection. After one cycle of pressurization (1 → 2 MPa), all membranes became more stable in rejecting NaCl in the second and third 1 MPa steps, exhibiting constant rejection values.

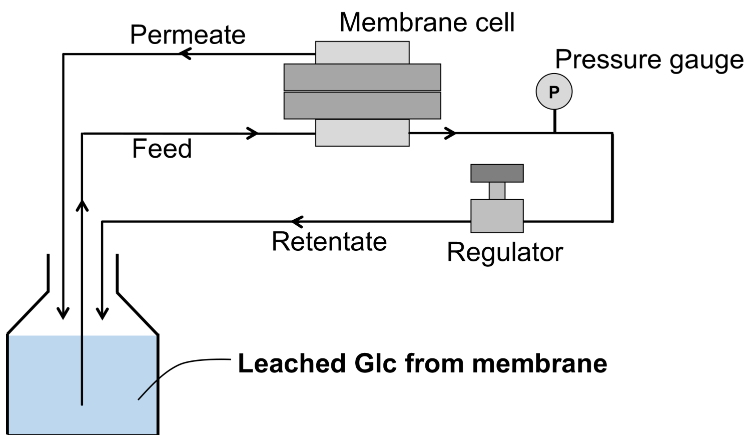

Supplementary Fig. 20. Schematic diagram of apparatus setups for measuring leached Glc amount from the membrane during the permeation test.

Ultrapure water with a known volume was used as the feed to measure the amount of leached Glc during the permeation test. Pressures of 1 and 2 MPa were successively applied for the permeation test with a duration of 12 h for each pressure condition, and the retentate and permeate solutions were reintroduced to the feed tank. After the permeation test, the TOC of the solution was measured to determine the amount of Glc leached from the membrane. Control permeation tests for pristine GO membranes were also performed to exclude the leached TOC from materials other than Glc in the membranes.

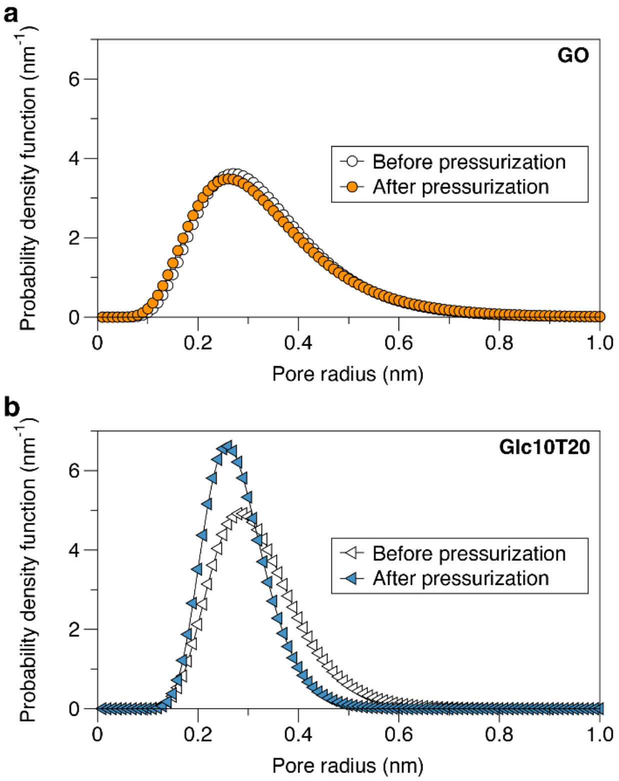

Supplementary Fig. 21. Fitted pore radius distribution of (a) GO and (b) Glc10T20 membranes.

The pore radius distribution curve was expressed as a probability density function.[1] The mean pore radius of the membrane was assumed to be the Stokes radius of the organic solute with a measured rejection of 50%. The geometric standard deviation of the distribution is assumed to be the geometric standard deviation of the probability density function curve, which is the ratio of the Stokes radius with a rejection of 84.13% to that with a rejection of 50%. The pore radius distribution of the membrane can thus be expressed as:

$$\frac{dR(r_p)}{dr_p} = \frac{1}{r_p ln\sigma_p \sqrt{2\pi}} exp\left[-\frac{(lnr_p - ln\mu_p)^2}{2(ln\sigma_p)^2}\right]$$

where r_p denotes the Stokes radius of the organic solute, σ_p the geometric standard deviation of the distribution curve, and μ_p the mean pore radius.

The Stokes radii of organic solutes can be calculated as

$$ln(r_p) = -1.4962 + 0.4654\ln(M_w)$$

where M_w denotes the molecular weight of the organic solute.

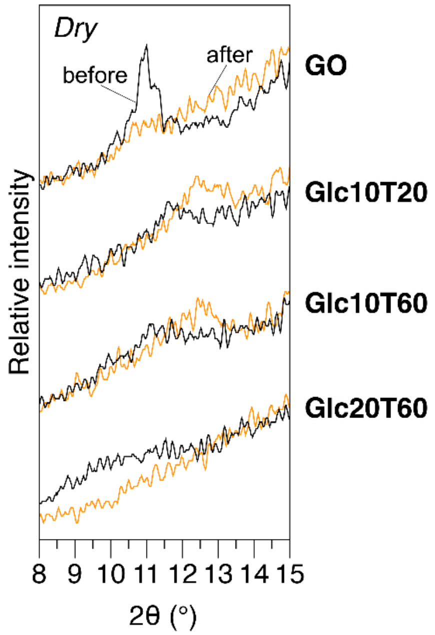

Supplementary Fig. 22. XRD spectra of the GO, Glc10T20, Glc10T60, and Glc20T60 laminates before and after the pressurized filtrations.

XRD spectra were obtained for the laminate samples before and after continuous pressurized water filtration under pressures of 1 and 2 MPa for a duration of 12 h for each pressure condition.

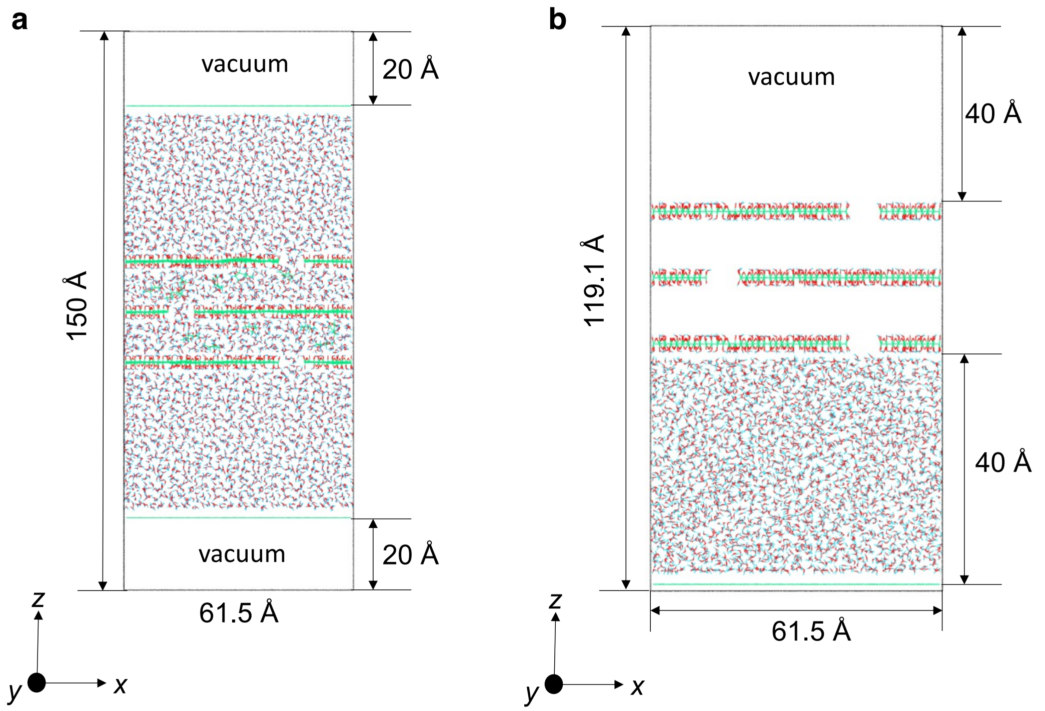

Supplementary Fig. 23. Boundary constraints applied for studying the (a) laminate structure stability, and (b) water transport.

Supplementary Tables

Supplementary Table 1. Summary of characteristic peak positions and corresponding d-spacings from the XRD analysis. The data for the Glc10T80 and Glc20T60 samples are not provided because their peak intensities were too low to be assigned.

Laminates		Peak position (2θ, °)			d-spacing (Å)		
		dry	wet	wet (NaCl)	dry	wet	wet (NaCl)
	GO	11.0	7.0	7.2	8.04	12.62	12.27
T'y'	Glc10T0	11.3	7.2	7.2	7.82	12.27	12.27
	Glc10T10	11.5	7.2	7.8	7.69	12.27	11.33
	Glc10T20	11.6	11.5	8.5	7.62	7.69	10.39
	Glc10T40	11.8	11.6	10.0	7.49	7.62	8.84
	Glc10T60	11.7	11.3	10.7	7.56	7.82	8.26
	Glc10T80	–	–	–	–	–	–
Glc'x'	Glc0T60	11.7	11.4	8.5	7.56	7.76	10.39
	Glc1T60	11.4	11.4	9.0	7.76	7.76	9.82
	Glc5T60	12.0	11.7	10.1	7.37	7.56	8.75
	Glc10T60	11.7	11.3	10.7	7.56	7.82	8.26
	Glc15T60	11.6	11.4	11.0	7.62	7.76	8.04
	Glc20T60	–	–	–	–	–	–

Supplementary Table 2. MWCO of different membranes before and after pressurization.

	**GO**	**Glc10T20**	**Glc10T60**	**Glc20T60**
MWCO before	480 Da	311 Da	180 Da	352 Da
MWCO after	>500 Da	188 Da	180 Da	>500 Da

Supplementary Table 3. Summary of literature reports on GO-based membranes exhibiting relatively high NaCl rejection.

Membrane code	Permeance [L m^{-2} h^{-1} bar^{-1}]	Feed NaCl concentration [ppm]	NaCl rejection [%]	Reference
PE@ArGO	2.9	58.4	88	2
GO-FLG	0.35	2000	85	3
GO (EPR)	25	500	97	4
GNM/SWNT	110.6	2000	85	5
TU-GO	1.5	100	95	6
TCPP-rGO	0.17	500–2000	85–92	7
Air-rGO	1.05	1000	83	8
K-rGO	0.6	1000	91	9
GO-TBO	0.4	584–29220	30–85	10
TA-rGO	0.36	500–2000	80–92	11
GO/g-C_3N_4	33.5	1170	87	12
GO-AQP	7.83	1000	99.1	13
**Glc-rGO (This study)**	0.14	500–5000	88.7–93.2	

Supplementary references

1. Liang Y*, et al.* Polyamide nanofiltration membrane with highly uniform sub-nanometre pores for sub-1 Å precision separation. *Nat. commun.* **11**, 2015 (2020).
2. Song X*, et al.* Charge-Gated Ion Transport through Polyelectrolyte Intercalated Amine Reduced Graphene Oxide Membranes. *ACS Appl. Mater. Interfaces* **9**, 41482-41495 (2017).
3. Morelos-Gomez A*, et al.* Effective NaCl and dye rejection of hybrid graphene oxide/graphene layered membranes. *Nat. Nanotechnol.* **12**, 1083-1088 (2017).
4. Li W, Wu W, Li Z. Controlling Interlayer Spacing of Graphene Oxide Membranes by External Pressure Regulation. *ACS Nano* **12**, 9309-9317 (2018).
5. Yang Y*, et al.* Large-area graphene-nanomesh/carbon-nanotube hybrid membranes for ionic and molecular nanofiltration. *Science* **364**, 1057-1062 (2019).
6. Yuan B*, et al.* Cross-linked Graphene Oxide Framework Membranes with Robust Nano-Channels for Enhanced Sieving Ability. *Environ. Sci. Technol.*, (2020).
7. Guan KC*, et al.* Nanochannel-confined charge repulsion of ions in a reduced graphene oxide membrane. *J. Mater. Chem. A* **8**, 25880-25889 (2020).
8. Yuan S*, et al.* Minimizing Non-selective Nanowrinkles of Reduced Graphene Oxide Laminar Membranes for Enhanced NaCl Rejection. *Environmental Science & Technology Letters* **7**, 273-279 (2020).
9. Yuan S, Li Y, Xia Y, Selomulya C, Zhang XW. Stable cation-controlled reduced graphene oxide membranes for improved NaCl rejection. *J. Membr. Sci.* **621**, 118995 (2021).
10. Wang ZZ, Ma C, Xu CY, Sinquefield SA, Shofner ML, Nair S. Graphene oxide nanofiltration membranes for desalination under realistic conditions. *Nature Sustainability*, (2021).
11. Guan K, Jia Y, Lin Y, Wang S, Matsuyama H. Chemically Converted Graphene Nanosheets for the Construction of Ion-Exclusion Nanochannel Membranes. *Nano Lett.* **21**, 3495-3502 (2021).
12. Wu Y*, et al.* 2D Heterostructured Nanofluidic Channels for Enhanced Desalination Performance of Graphene Oxide Membranes. *ACS Nano* **15**, 7586-7595 (2021).
13. Lee CS, Kim I, Jang JW, Yoon DS, Lee YJ. Aquaporin-Incorporated Graphene-Oxide Membrane for Pressurized Desalination with Superior Integrity Enabled by Molecular Recognition. *Adv Sci (Weinh)* **8**, e2101882 (2021).

Supplementary Movie 1 (separate file). Molecular dynamics simulation of the GO laminate structure under pressure.

Supplementary Movie 2 (separate file). Molecular dynamics simulation of the rGO laminate structure under pressure.

Supplementary Movie 3 (separate file). Molecular dynamics simulation of the Glc(2)-rGO laminate structure under pressure.

Supplementary Movie 4 (separate file). Molecular dynamics simulation of the Glc(6)-rGO laminate structure under pressure.

Supplementary Movie 5 (separate file). Molecular dynamics simulation of water molecule transport through GO laminates.

Supplementary Movie 6 (separate file). Molecular dynamics simulation of water molecule transport through Glc(6)-rGO laminates.
