# Supplementary material for: Deformation constraints of graphene oxide nanochannels under reverse osmosis
Source: Nat Commun. 2023 Feb 23;14:1016. doi: 10.1038/s41467-023-36716-5 (PMC9950365; doi:10.1038/s41467-023-36716-5)
Supplement: Supplementary file 3 — Description of Additional Supplementary Files [file 41467_2023_36716_MOESM3_ESM.pdf]

Description of Additional Supplementary Files

File Name: Supplementary Movie 1
Description: Molecular dynamics simulation of the GO laminate structure under pressure.

File Name: Supplementary Movie 2
Description: Molecular dynamics simulation of the rGO laminate structure under pressure.

File Name: Supplementary Movie 3
Description: Molecular dynamics simulation of the Glc(2)-rGO laminate structure under pressure.

File Name: Supplementary Movie 4
Description: Molecular dynamics simulation of the Glc(6)-rGO laminate structure under pressure.

File Name: Supplementary Movie 5
Description: Molecular dynamics simulation of water molecule transport through GO laminates.

File Name: Supplementary Movie 6
Description: Molecular dynamics simulation of water molecule transport through Glc(6)-rGO laminates.
